# The Impact of Maternal Folates on Brain Development and Function after Birth

**DOI:** 10.3390/metabo12090876

**Published:** 2022-09-16

**Authors:** Sapna Virdi, Nafisa M. Jadavji

**Affiliations:** 1 Biomedical Sciences Program, Midwestern University, Glendale, AZ 85308, USA; 2 College of Osteopathic Medicine, Midwestern University, Glendale, AZ 85308, USA; 3 College of Veterinary Medicine, Midwestern University, Glendale, AZ 85308, USA; 4 Department of Neuroscience, Carleton University, Ottawa, ON K1S 5B6, Canada

**Keywords:** maternal folate, folic acid, folate deficiency, brain, neurodevelopment

## Abstract

Folate is vital for biological processes within the body, including DNA synthesis, DNA repair, and methylation reactions that metabolize homocysteine. The role of folate is particularly important in pregnancy, where there is rapid cellular and tissue growth. Maternal folate deficiencies secondary to inadequate dietary supplementation are known to produce defects in the neural tube and spinal cord, yet the exact mechanism of folate in neurodevelopment is unknown. The consequences of maternal folate deficiency on offspring brain development and function beyond gestation are not well defined. The objective of this review is to investigate the role of folate deficiency in offspring neurodevelopment, and the complications that arise post-gestation. This was accomplished through a comprehensive review of the data presented in both clinical and preclinical studies. Evidence supports that folate deficiency is associated with altered offspring neurodevelopment, including smaller total brain volume, altered cortical thickness and cerebral white matter, altered neurogenesis, and neuronal apoptosis. Some of these changes have been associated with altered brain function in offspring with memory, motor function, language skills, and psychological issues. This review of literature also presents potential mechanisms of folate deficiency in neurodevelopment with altered metabolism, neuroinflammation, epigenetic modification through DNA methylation, and a genetic deficiency in one-carbon metabolism.

## 1. Introduction

**Figure 1:**
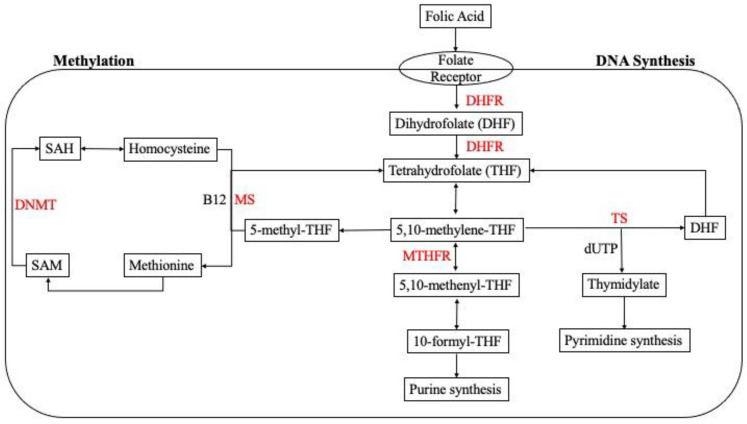
Cellular one-carbon (1C) metabolism. Folic acid undergoes a series of enzymatic reactions in the cell for purine and pyrimidine synthesis in the folate cycle. Homocysteine is metabolized through re-methylation in the methionine cycle. Abbreviations: 5,10-methylene-THF: 5,10-methylene-tetrahydrofolate; 5,10-methenyl-THF: 5,10-methenyl-tetrahydrofolate; 10-formyl-THF: 10-formyl-tetrahydrofolate, 5-methyl-THF: 5-methyl-tetrahydrofolate; SAM: S-adenosylmethionine; SAH: S-adenosylhomocysteine; DHFR: dihydrofolate reductase; MTHFR: methylenetetrahydrofolate reductase; TS: thymidylate synthase; MS: methionine synthase; DNMT: DNA methyltransferase.

Folate, also known as Vitamin B9, is a water-soluble nutrient that is found naturally in food sources. Folate plays a vital role in one-carbon metabolism as a substrate for purine and pyrimidine synthesis, DNA repair, methylation reactions, and amino acid homeostasis [1] (Figure 1). Folic acid is the chemically synthesized form of folate that is enriched in food and supplements [2]. In the cell, both folate and folic acid undergo a series of conversion reactions to produce 5-methyltetrahydrofolate, which is the biologically active form that is utilized in cellular processes [3]. Folate demand in the body increases with pregnancy secondary to support rapidly growing maternal and fetal tissue. Current recommendations for folic acid supplementation in pregnancy are 400 μg/day for women who are planning to become pregnant [4]. Recommendations for folic acid supplementation during pregnancy are 600 μg/day and 500 μg/day for the duration of lactation [5].

Folate deficiency in pregnancy, which is typically classified as any serum value below 2 ng/mL, occurs as a result of insufficient dietary intake of folic acid [6]. Maternal folate deficiency is known to alter neurodevelopment and produce neural tube defects such as anencephaly, hydrocephalus, and spina bifida [7], though the mechanism behind these are not well defined yet. The impact of folate deficiency post-gestation has not been well studied. The objective of this review is to determine the role of maternal folate levels during pregnancy and lactation on offspring neurodevelopment after birth. This review presents clinical (Table 1) and preclinical literature (Table 2) to describe the outcomes and mechanisms of maternal folate deficiency on offspring neurological function.

## 2. The Impact of Maternal Folate Levels on Offspring Neurological Function in Clinical Populations

Clinical and preclinical studies have provided evidence that maternal folate deficiency can produce complications in offspring, such as altered brain development and morphology, including smaller brain size [18] and decreased thickness of specific brain regions [16]. Clinical work has also shown that maternal folate status can alter neurodevelopment in offspring and play a role in neuropsychological disorders (Table 1) [13].

Adequate folic acid supplementation prenatally and through gestation is positively associated with child neurodevelopment [12]. A study based on data derived from the ‘Rhea’ mother-child cohort showed that a daily maternal dietary supplementation of 5 mg folic acid during early pregnancy resulted in improved neurodevelopment when evaluated at an age of 18 months in the offspring [13]. Neurodevelopment assessment was based on psychomotor and mental scales in the offspring. Children who had high folic acid exposure (5 mg/day) in early pregnancy had a 5-unit increase in receptive communication (language scale), which is comprised of preverbal behavior, and vocabulary development, and verbal comprehension, when compared to no folic acid exposure. There was also a 3.5-unit increase in expressive communication (language scale), which included preverbal communication, vocabulary development, and morpho-syntactic abilities when compared to no maternal folic acid exposure. However, there was no positive relationship between neurodevelopment and excessive folic acid exposure (greater than 5 mg/day).

Neurodevelopment was assessed in 2-year-old children within the Maternal Key Nutritional Factors and Offspring’s Atopic Dermatitis (MKFOAD) birth cohort [17]. The mothers had serum folate concentrations measured at 12–14 weeks gestation (early pregnancy), 22–26 weeks gestation (mid pregnancy), and 34–36 weeks gestation (late pregnancy). The children’s neurodevelopment was examined via the Gesell Development Scale, which tests for gross and fine motor, language, adaptive, and social behavior. A significant correlation was found between maternal folate status in late pregnancy (34–36 weeks gestation) and neurodevelopment. For each 10 nmol increase in serum folate concentration, there was a 3.1-unit increase in the language developmental quotient of children.

Similarly, results from a population-based cohort in Spain showed that children had improved neurodevelopment associated with gestational folic acid supplementation when assessed at age 5 [9]. The mothers included in this study either had no folic acid supplementation, direct folic acid supplementation, or supplementation through multivitamins by the end of the first trimester of pregnancy. However, information regarding folic acid dosage and exact timing when supplementation began was not available. Five-year-old children who had folic acid exposure during gestation scored higher for verbal, motor, and verbal executive functions, as well as for assessments for social competence and inattentive symptoms when compared to no maternal folic acid supplementation.

The effect of maternal folate status on child psychological health was presented in work embedded within the ‘Generation R Study’ [14]. Children at the age of 3 were assessed for emotional issues, including being emotionally reactive, anxious, or depressed, as well as behavioral issues identified as aggressive behavior or problems with attentiveness. Children of mothers with folate deficiency in early pregnancy (less than 10 weeks gestational or preconceptionally) had a higher risk for emotional issues, but not behavioral issues. There was also an increased risk for the development of emotional problems in the offspring of mothers who did not take any folic acid supplementation or who started late in pregnancy, which was considered inadequate folic acid supplementation.

Data from the ‘Generation R Study’ was also used to determine whether prenatal folate exposure affected long-term brain development in children ages 9–11 [32]. Maternal blood folate levels were related to brain structure and development via magnetic resonance imaging (MRI). The neuroimaging showed that there was smaller total brain volume and cerebral white matter in offspring aged 9–11 when compared to adequate prenatal folic acid exposure. Further, the relationship between brain morphology and development with emotional and behavioral problems was investigated. The children’s mothers were given a child behavioral assessment when the children were aged 10, to assess for emotional and/or behavioral issues. There was no association between child emotional or behavioral problems with maternal folate levels. However, it was noted that psychological issues in children typically develop much later than at 10 years of age.

Prenatal folic acid supplementation and its role in severe language impairment and delay was studied in 3-year-old children in the ‘Norwegian Mother and Child Cohort Study’ [14]. Maternal folic acid supplementation was classified into two groups: supplementation starting 4 weeks prior to conception, or supplementation starting at 8 weeks after conception. Severe language delay for this study was defined as speaking only one word or unidentifiable words at the age of 3. Language skills were determined through a questionnaire using a 6-point grammar scale that was administered by the mothers. The main findings of this study revealed that folic acid supplementation from 4 weeks preconception to 8 weeks post-conception significantly reduced the risk of severe language delay. However, the women who began folic acid supplementation after week 8 did not have a reduced risk of severe language delay. Motor skills were also evaluated through the ‘Ages and Stages Questionnaire’ that was administered to the mothers as well. The results showed that there was no relationship between a delay in motor skills and folic acid supplementation in children up to the age of 3 years.

A positive association was discovered between maternal folate supplementation and emotional intelligence and resiliency tests in children [15]. Pregnant women who had taken the recommended folic acid supplementation (400 μg/day) in the first trimester were included in this study. The participants either continued the recommended dose throughout the entire pregnancy, or were given a placebo from the second trimester onward. Children who were exposed to full-term supplementation of folic acid scored higher on both the emotional intelligence (emotional knowledge, control, and response) and resiliency (overcoming adverse situations) tests when compared to those who were exposed to folic acid in the first trimester only. Using regression analysis, the researchers determined that maternal folate status at 36 weeks gestational age was an indicator for the development of emotional intelligence and resiliency.

Maternal folate status was correlated with children’s brain growth and potential consequences on behavior at the age of 9 [10]. Red blood cell folate was measured early in pregnancy (only at 14 weeks) in the mothers, followed by total folate intake measurements in both the early and late stages of pregnancy. Lower red blood cell folate and total folate intake levels in early pregnancy were correlated with higher levels of hyperactivity (overactive, distracted, and fidgeting) and peer problems (being bullied, unliked by peers, and playing alone). There was no association found with emotional (unhappy, nervous, or worried) or conduct (bullying peers, disobedient, or lying) problems. It was also noted that there was no association between total folate intake in later pregnancy and hyperactivity or peer problems (red blood cell folate was only measured in early pregnancy). Maternal red blood cell folate status was positively correlated with measures of head circumference. This suggested that folate status during gestation impacts on brain development, and that behavioral issues may be secondary to altered neurodevelopment.

Fetal folic acid exposure and its relationship to neurodevelopment and psychiatric health was assessed in children ages 8 to 18 years [16]. The subjects for this study were derived from three different cohorts, including a clinical cohort at the ‘Massachusetts General Hospital’ (MGH), and two community cohorts, identified as the ‘Philadelphia Neurodevelopmental Cohort’ (PNC) and the ‘National Institutes of Health Magnetic Resonance Imaging Study of Normal Brain Development’ (NIH). The MGH cohort included participants that were born either 3.5 years before or after folic acid fortification was introduced in the US, the PNC cohort, with participants who born throughout the introduction of folic acid fortification, and those in the NIH cohort, who were born before the fortification was initiated. Cortical thickness changes were assessed in the participants through MRI, and compared amongst groups that had absent, partial, or adequate fetal folic acid exposure. In the MGH cohort, participants who had adequate prenatal folic acid exposure had increased cortical thickness compared to the group with no folic acid exposure. It was noted that the group with partial folic acid exposure had increased cortical thickness when compared to the participants with no exposure; however, cortical thickness was less than the group with adequate exposure.

When investigating specific age-related changes in cortical thickness, it was found that between the ages 8 to 18, there was increased thickness in the bilateral frontal cortex and the right inferior temporal gyrus of the children who were exposed to folic acid. There was also delayed age-associated cortical thinning in the left inferior temporal and left inferior parietal regions in the children who had full exposure to folic acid vs. the non-exposed. Similar results were found in the PNC cohort, where folic acid supplementation delayed cortical thinning in the left frontal, right inferior temporal, and left and right inferior parietal regions. It was estimated that the cortical thinning occurred between the ages of 13 and 14.3 years in these children. However, for the NIH cohort, which included participants who were born before the implemented folic acid fortification, cortical thinning was only found in the left frontal cortex of the participants. It was also noted here that the cortical thinning occurred at an average of 11.87 years old, which was younger than the findings in the PNC cohort. In terms of psychological health, only the PNC cohort had adequate clinical data available for assessment. It was found that the occurrence of psychosis symptoms increased with levels of cortical thinning in the frontal, temporal, and parietal regions. This finding was presented as a probable mechanism for neuropsychiatric symptoms in the participants. The discussion of potential mechanisms of folic acid and the effect on neurodevelopment is discussed next.

## 3. Folate Mechanism of Action

Folate has a vital role in cell metabolism, where it functions as a cofactor in purine and pyrimidine synthesis, DNA repair, and methylation reactions that alter gene expression [1]. In pregnancy, folate demand increases secondary to cell division and the growth of fetal and maternal tissue. Maternal folate deficiency is known to cause neural complications in utero and post-gestation, although the mechanism of action has not been identified. In this section, the potential mechanisms that may drive neurological changes in offspring brain tissue as a result of maternal folate dietary deficiencies will be discussed. These studies are also summarized in Table 2.

### 3.1. The Impact of Folate Deficiency on Cortical Morphology and Neurogenesis

Folate deficiency has been shown to alter cortical neurodevelopment in mice, and a study also found that excessive maternal folate supplementation altered neurodevelopment, with potential consequences for neurobehavior [28]. For this study, folate deficiency was defined as 0 mg/kg folic acid and excess as 20 mg/kg folic acid, which was supplemented in the pregnant dam’s diet. It was found that both folic acid deficiency and excess supplementation resulted in altered levels of early-born deep layer and later-born upper layer neurons when tested in the mice brain at post-natal day 0. Specifically, the levels of late-born neurons and brain-2 gene (Brn2) were increased, and the levels of early-born neurons, T-brain-1 (Tbr1) and chicken ovalbumin upstream promoter transcription factor interacting protein 2 (Ctip2) were reduced at similar levels in both folic acid deficiency and excess. To determine a potential mechanism for the altered neurogenesis, it was found that radial glial cells were delayed in transitioning to intermediate progenitors in early neurogenesis. Further, the levels of deep layer neurons increased to a greater extent as a result of increased apoptosis, which was represented by both folic acid deficiency and excess. To determine whether these cortical morphological alterations affected behavior, 4–6-week-old mice were tested via behavioral assays that showed increased levels of anxiety in the folate excess supplemented mice compared to the control (2 mg/kg folic acid), although there was no difference in the deficient group when compared to the control. The folate-deficient mice underperformed in tests for short-term memory when compared to the control, but there were no changes for the excess group. Overall, it was concluded that alterations in cortical morphology affected mice neurobehavior negatively for both the folate-deficient and -excess groups.

### 3.2. Altered Neurogenesis and Apoptosis in the Fetal Brain

A study performed in mice showed that folate deficiency during late gestation resulted in decreased neural progenitor cells in the fetal brain, specifically, 47% in the septum, 43% in the caudate putamen, and 54% in the neocortex [19]. Additional findings included increased levels of apoptosis in the fetal brain septum of folate-deficient mice when compared to folate-supplemented mice, which also contributed to neural progenitor cell loss. This work was a continuation from a prior study that showed calretinin, which is a calcium binding protein expressed in the neurons of the ventral forebrain (role in memory, sleep, and attention) throughout life, was increased in choline-deficient mice. Similarly, the folate-deficient mice were also found to have an increased expression of calretinin in the forebrain when compared to folate-supplemented mice, suggesting that maternal folates have a role in neurodevelopment.

### 3.3. Neuroinflammatory Response to Folate Deficiency and Altered Behavior

Gestational folic acid supplementation and its effect on memory and neural health was tested in female rats [30]. The rats were either exposed to a control diet (AIN-93), a folate-deficient diet, or a diet containing 5, 10, or 50 mg/kg folic acid. The mice were tested at 2 months (young) and 18 months (aged) via behavioral tests for memory assessment. Neural health, specifically neuroinflammation, was tested via the detection of brain derived neurotrophic factor, nerve growth factor, TNF-α, IL-1β, and IL-4. Behavioral testing showed that the 18-month-old mice (aged) with deficient gestational folic acid had aversive, spatial, and habituation memory loss. These mice also had increased hippocampal levels of TNF-α and IL-1β, which are present with aging. However, lower levels of these cytokines were present in the 18-month-old mice who did have exposure to gestational folic acid. Similarly, levels of IL-4 were protected by folic acid supplementation in the aged mice, as levels of IL-4 were found to be decreased in the control aged mice. In the young mice who were folic acid-deficient, IL-4 levels were decreased, and along with aging, the levels of hippocampal brain derived neurotrophic factor and prefrontal cortex nerve growth factor were also decreased. However, in the mice that received folate supplementation, these levels were protected.

### 3.4. Metabolic and Proteomic Changes in Response to Folic Acid Supplementation

The effect of folic acid supplementation on neural development was investigated through metabolic changes in female rat offspring [29]. The offspring were either exposed to 2 mg/kg folic acid (control) in gestation or 5 mg/folic acid (experimental). Blood samples were collected at weeks 0, 3, and 7, along with brain tissue samples at week 7. The results showed that there were increased levels of global methylation in the liver and adipose tissue of the offspring that were exposed to maternal folic acid supplementation. In terms of metabolic changes, levels of phosphatidylcholine and lysophosphatidylcholine were found to be increased in the offspring that received folic acid supplementation, indicating that folic acid may have a role in lipid metabolism. Similarly, the levels of docosahexaenoic acid were increased, and free fatty acids were decreased, including C16:0, C18:1, C18:2, ƴ-C18:3, C20:4, and C22:4, in offspring with folic acid supplementation, suggesting that there may a role in fatty acid metabolism as well. Metabolites that are known to be associated with neural health were also altered in the folic acid-supplemented group, including increased ƴ-aminobutyric acid (GABA), glycine, tryptophan, serine, and methionine. These changes reflect the role of folic acid in DNA methylation, as these metabolites are involved in methylation. This finding was relative to increased levels of global methylation found in the liver and adipose tissue of folate-supplemented offspring. Three neuronal-related proteins were also upregulated in folate-supplemented offspring, including G-protein, which is known to be associated with neurotransmitters such as GABA, as well as CAMK2G and PPP2R1B, with both having a potential role in memory and learning. Behavioral testing was performed at week 7 to test memory and learning in the offspring. It was shown that the offspring exposed to folic acid had improved memory skills when compared to the offspring that had no supplementation. These metabolic and proteomic changes were introduced as a potential mechanism for folic acid’s role in neurodevelopment.

### 3.5. The Role of Folate Deficiency in Epigenetic Modification: Methylation

DNA methylation and maternal folate status during pregnancy and lactation was investigated in adult female mice [20]. Specifically, this study explored the role of folate deficiency in DNA methylation and DNA repair through base excision repair activity. The offspring were either exposed to 2 mg/kg folic acid, which was considered to be adequate folate supplementation, or 0.4 mg/kg folic acid, which was folate deficient, during pregnancy and lactation. At postnatal day 22–25 (weaning), the offspring were started on a control diet of 5% anhydrous milk fat, or a high-fat diet with 20% anhydrous milk fat. At 6 months of age, the offspring were sacrificed to collect cerebellar, hippocampal, cortical, and subcortical brain tissue. The results showed that low folate supplementation and a high-fat diet at weaning resulted in decreased base excision repair activity in all four brain regions of the offspring. This was hypothesized to be secondary to decreased nucleotide availability, as folate plays a role in nucleotide synthesis. Levels of oxidative damage were also found in the brain of offspring who were exposed to a folate-deficient diet, and this was measured through levels of 8-oxodG. Based on these results, it was proposed that folate deficiency and a high-fat diet can lead to increased oxidative damage and altered levels of base excision repair, which have a role in neurological disorders.

Gene expression in the fetus and the effect of folic acid supplementation on DNA methylation was studied in rats [23]. This study primarily focused on the gestational period in which the organs were more sensitive to folic acid supplementation. Male and female rats at 6 weeks of age were placed on a classified amino acid diet with a 2 mg/kg folic acid diet for 2 weeks prior to mating. After the confirmation of pregnancy, the dams were placed on five different folic acid diets: control with 2 mg/kg folic acid, 2.5× folic acid in the control diet for either the first, second, or third weeks of gestation, or 2.5× folic acid in the control diet throughout the entire pregnancy. At birth, the pups were sacrificed to collect blood and plasma for folate and homocysteine quantification, as well as tissue samples of the brain, liver, kidney, and colon, to assess DNA methylation and gene expression. The results showed that pups had 30–42% higher folate concentrations when compared to no folate supplementation. Specifically, brain folate concentration increased the most in the pups when folic acid was supplemented during the second and third weeks of gestation. Additionally, liver folate concentrations were noted to increase throughout the entire pregnancy instead of during a specific gestational period. DNA methylation decreased by 18% in the brain when supplementation was started in the second week of gestation, but there was no significant effect noted at one week. Methylation levels also decreased in the liver throughout pregnancy instead of during a specific time period, and the kidney and colon were not affected by supplementation. Folic acid supplementation in late gestation decreased the gene expression of Er-α and pPAR-α by 15–25% in the liver; however, the brain, kidney, and colon did not have altered gene expression.

Methylation of the insulin-like growth factor 2 (IGF2) gene was investigated in children aged 17 months within the ‘HAVEN’ study to determine if periconceptional folic acid status affected DNA methylation [8]. IGF2 was targeted for this study, as it is a growth factor known to be affected by periconceptional folic acid, and is expressed in most embryonic tissues (in mice). Birth weight association with maternal folic acid exposure and IGF2 methylation was also explored to determine if there were any correlated phenotypic effects. To explore the role of methylation, 5 CpGs in the IGF2 differentially methylation region (DMR) were specifically targeted. Methylation was measured in blood samples obtained from the mothers and their 17-month-old children. It was discovered that IGF2 DMR methylation increased by 4.5% in children who had periconceptional folic acid exposure (400 μg/day 4 weeks before conception and 8 weeks after), compared to those that did not have folic acid exposure. Additionally, maternal folic acid supplementation did not alter the levels of S-adenosylmethionine or S-adenosylhomocysteine in the mothers or children. However, it was noted that the levels of S-adenosylmethionine in the mothers correlated with levels of IGF2 DMR methylation in the offspring. Further, birth weight was correlated to IGF2 methylation, and it was discovered that birth weight decreased as the levels of methylation increased. There was no correlation discovered between periconceptional folic acid exposure and birth weight. Overall, it was concluded from these findings that periconceptional folic acid exposure have a role in epigenetic modification in offspring.

Genome-wide differential DNA methylation and the potential association with maternal plasma folate were evaluated in both the ‘Generation R Study’ and the ‘Norwegian Mother and Child Cohort study’ [14]. For this study, cord blood was quantified for DNA methylation from children in both birth cohorts, and was analyzed with maternal folate levels. Genome-wide analysis revealed that 443 CpGs or 320 genes are associated with plasma folate levels during pregnancy. Some of these genes have more than one identified CpG and are known to have a role in development, neural health, and neurological disorders. These genes include adenomatosis polyposis coli 2 gene (APC2), which is found in the human fetal and adult brains [33], LIM homeobox 1 (LHX1) with a role in uterine [34] and retinal development [35], Indian hedgehog (IHH) in skeletal malformation, roundabout guidance receptor 3 (ROBO3) in horizontal gaze palsy, glutamate metabotropic receptor 8 (GRM8), which has a role in ADHD [33], opioid-binding protein/cell adhesion molecule like (OPCML) and peripherin (PRPH) [36,37] in amyotrophic lateral sclerosis, and CUB and sushi multiple domains 1 (CSMD1) in schizophrenia and autism. The identification of these genes in relation to maternal folate levels signifies a role in neurodevelopment and potential neural disorders.

### 3.6. Altered Neurodevelopment and Neurobehavior

The duration of folic acid supplementation and its effect on neurodevelopment was tested in rat offspring [26]. The offspring were either exposed to a normal folate diet (2.1 mg/kg), a folate-deficient diet (0.1 mg/kg), or a folate short-term diet (3.5 mg/kg folic acid) that was started at mating, continued for 10 consecutive days, and then stopped to introduce the normal folate diet, as well as a folate long-term diet (3.5 mg/kg). Neurobehavioral testing performed at postnatal days 4 and 8 showed that the offspring that were exposed to folate during gestation had improved sensory-motor development. This was represented by scoring in the surface lighting reflex test and geotaxis testing. The Morris Water Maze test was administered for memory and learning assessment at postnatal day 45 for adolescent offspring and at postnatal day 90 for adult offspring. The folate-deficient group showed increased time in escaping the maze when compared to the folate-supplemented groups. However, the folate long term-supplemented group showed improved performance throughout the testing when compared to the folate short-term group. Overall, it was concluded that folate supplementation improved neurobehavior when compared to a folate-deficient diet during gestation. However, a folate diet that continued throughout pregnancy was noted to be more beneficial than supplementation in the preconception phase only. Additionally, hippocampal structure was assessed in the adult offspring. The adult offspring that were folate deficient showed fewer organelles and altered cell bodies (condensed chromatin), as well as altered mitochondria, rough endoplasmic reticulum, and Golgi body structure. These changes were not evident in the folate long term-supplemented group. Altered axon and synapse myelination were also discovered in the folate-deficient group, with the swelling of capillaries. This was also prevented in the folate long term-supplemented group.

Folate deficiency and its role in brain development postnatally was tested in rats during the weaning (postnatal day 10 and 23) and the post-weaning stage from postnatal day 40–70 [21]. Pregnant dams were fed a diet with 2 mg/kg folic acid and after birth, and the pups were either continued on the folate-supplemented diet or a diet with no folic acid. On postnatal days 10 and 23 (weaning), the pups were sacrificed for liver and brain assays to quantify folate and homocysteine levels. The dams were also sacrificed on day 23 for these assays. Behavioral testing of the pups was performed on postnatal days 40–70 (post-weaning), and the pups were sacrificed following this. Behavioral testing included an open field test to assess for exploratory behavior, locomotor activity, and anxiety, as well as place avoidance tests to analyze memory and cognition in the pups. The biological assays showed that the weaning folate-deficient pups had lower hepatic folate concentrations compared to the pups who had adequate weaning folate supplementation. Hepatic homocysteine concentrations were also higher in the weaning folate-deficient group. It was noted that there was no difference in brain folate concentrations amongst the weaning folate supplemented vs. non-supplemented group, although homocysteine levels were higher in the brain of the folate-deficient pups, specifically in the hippocampus and cerebellum. Behavioral testing revealed the weaning folate-deficient pups had impairments in spatial learning and long-term memory when compared to the folate-supplemented pups. It was noted that folic acid supplementation in the post-weaning stage did not change the neurobehavioral deficits present in the weaning folate-deficient pups, although gestational supplementation was adequate.

### 3.7. MTHFR Deficiency and Hippocampal Structural Change

Methylenetetrahydrofolate reductase (MTFHR) is an enzyme that converts 5,10-methylenetetrahydrofolate to 5-methyltetrahydrofolate, which is the folate source for methylation [38]. An MTHFR variant identified as 677TT (from 677C) is found in 20–40% of individuals in the United States [39]. MTHFR mutations are known to cause increased homocysteine levels, which can result in neural tube defects [40]. Maternal MTHFR deficiency and maternal dietary folate and choline deficiency was studied in 3-week-old mice to determine the effect on offspring brain health and function [22]. Short-term memory was tested in these mice using the novel object recognition task (short-term memory) and the Y-maze test (spatial short-term memory). It was found that maternal MTHFR-deficient offspring had short-term memory impairment. Hippocampal tissue was also tested in the offspring for cell death and structural changes. The offspring from the MTHFR-deficient mothers were noted to have increased hippocampal cell death. Maternal folate and choline dietary deficiency also resulted in short-term memory impairment and increased hippocampal apoptosis. A proposed mechanism for both memory and hippocampal tissue changes was increased maternal homocysteine levels, which alter one-carbon metabolism.

## 4. Discussion

**Figure 2:**
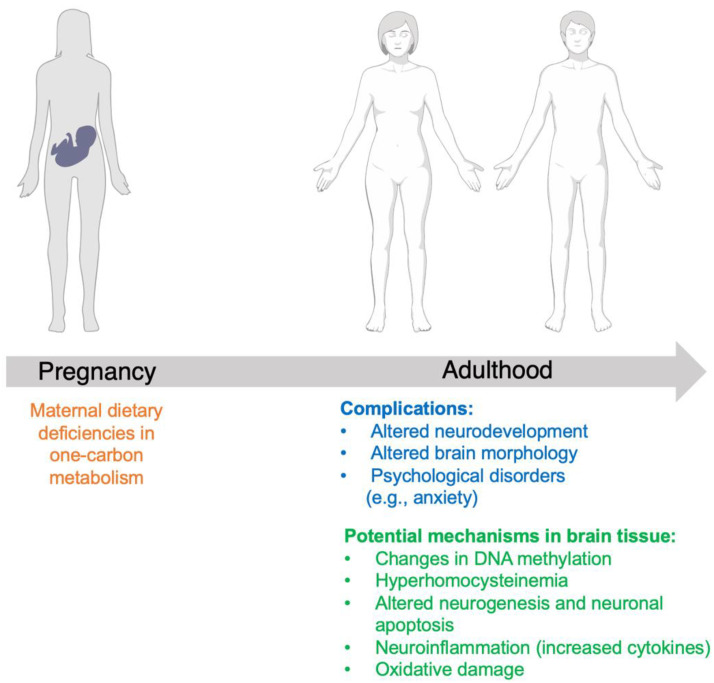
Summary of complications in offspring secondary to maternal dietary folate deficiency, and potential mechanisms of folate in neurodevelopment.

The importance of maternal folate supplementation is well understood, as is the role of folate deficiency in offspring neural tube defects, and it has been shown to improve brain function postnatally [41]. However, folate’s mechanism of action has not been established, nor has the effect of maternal folate deficiency on offspring after gestation. Maternal folate deficiency can be a result of diet or genetics factors (e.g., MTHFR deficiency). Through a comprehensive review of preclinical and clinical data, this review presents the complications of maternal folate deficiency beyond gestation, as well as the potential mechanism of action for folic acid in neurodevelopment; the results of our review of the literature are summarized in Figure 2. Each study we reviewed had a limitation; these are listed in detail within Table 1 and Table 2. It is important to note that folic acid interacts with other biomolecules and micronutrients, and it can be hard to determine the role that folic acid plays in physiological functions [42]. Evidence suggests that there is a positive association between maternal folate supplementation and neurodevelopment, particularly if supplementation is started preconceptionally or in the early weeks of gestation. Improved neurodevelopment in children has been associated with improved language scales, motor function, social competence, improved emotional intelligence and resiliency, decreased risk for behavioral and emotional issues, decreased hyperactivity and peer problems, and improved memory. Clinical data indicate that maternal folate deficiency can result in smaller total brain volume and cerebral white matter, as well as altered cortical morphology, neurogenesis, and neuronal apoptosis. Proposed mechanisms for altered neurodevelopment in response to maternal folate deficiency are based on preclinical data, and include altered lipid and fatty acid metabolism, increased cytokines and neuroinflammatory response, DNA methylation altering gene expression, and oxidative damage; and maternal MTHFR deficiency results in hyperhomocysteinemia.

## 5. Future Directions

Future directions include highlighting the impact of excessive maternal folic acid supplementation on offspring, as some studies have shown negative results. A study performed in mice showed that a 10-fold increase in folic acid supplementation during pregnancy resulted in an altered expression of imprinted genes and transcriptional factors in the cerebellum of offspring [24]. Another study investigated the role of high folate intake during pregnancy on MTHFR deficiency, and the outcome on offspring [25]. The results suggested that high folate intake causes pseudo-MTHFR deficiency, and altered choline and methyl metabolism, as well as delays in embryonic development and impaired memory in the offspring. Excess folic acid supplementation (2.5-fold) prior to conception, during pregnancy, and during lactation were shown to increase levels of phosphorylated β-catenin in the brains of weaning and adult mice, which was introduced as a potential mechanism of folic acid in neurodevelopment [27]. Early exposure to excessive folic acid in pregnancy was shown to increase anxiety, and alter motor coordination and spatial memory in mice [31]. Future clinical studies may complement these findings in preclinical studies. In countries with mandatory folic acid fortification, the emergence of excessive supplementation with folic acid has occurred [43]. Future work investigating the role of excessive maternal folic acid on offspring neurological function in a human population needs to be conducted, as the impact on neural development in children is not known. Studies investigating paternal folate status at conception may also present potential mechanisms of folate function and outcomes in offspring.

## Figures and Tables

**Table 1:** Summary of main findings from clinical studies.

Citation	Study Question(s) Being Investigated	Study Population	Study Design	What Is the Setting of This Study	Describe the Main Findings	Limitations
Steegers-Theunissen [8]	Does periconceptional maternal folic acid effect IGF2 DMR methylation in offspring? Does this effect intrauterine growth?	Mother-child pairs chosen from an established study: ‘Haven’	Mother and children between ages 12 and 18 months were enrolled into the study in the Netherlands between October 2003 and January 2007.	Unknown location in Rotterdam, The Netherlands	Mothers who used folic acid had children with a 4.5% increase in *IGF2* DMR methylation, and *IGF2* DMR methylation levels in children were associated with *S*-adenosylmethionine blood levels of the mother. An inverse independent relationship between methylation of *IGF2* DMR and birth weight was reported.	Small sample size and recruitment of participants for from an already established birth cohort.
Julvez [9]	Does maternal folic acid supplementation alter neurodevelopment?	Mother-child pairs from an established birth cohort	The participants for this study were identified from a birth cohort in Spain. The study included women who had natal care from 12 weeks of gestation through birth, and the children were followed until 4 years of age.	Unknown specific location in Menorca, Spain	Folic acid supplementation in pregnancy improves cognition, motor skills, social skills, and attentiveness in 4-year-old children. These results were also adjusted for socioeconomic factors.	Researchers not able to validate self-reported folic acid supplementation and did not factor psychosocial variables.
Schlotz [10]	Is there an association between maternal folate status and brain growth and childhood behavioral problems?	Mother-child pairs from a previous nutrition during pregnancy and fetal growth study	The mothers and children (mean age 9 months and 8 years old) were recruited in the original study were in Southampton, UK.	Unknown location in Southampton, UK	Lower maternal folate status and total folate intake correlate with childhood hyperactivity and peer problem scores in offspring. Head circumference at birth is correlated with maternal folate status and that there was an inverse relationship with hyperactivity and peer problems.	Socioeconomic variable which may alter offspring neurodevelopment outside of dietary folic acid deficiency.
Roth [11]	Does prenatal folic acid supplementation affect the risk of developing severe language delay in offspring?	Mother-child pairs from the ‘Norwegian Mother and Child Study’	Maternal folic acid use for this study was defined as supplementation 4 weeks before conception and 8 weeks after conception. Children aged 3 years old at time of assessment.	Unknown specific location in Norway	Folic acid supplementation from 4 weeks prior to conception to 8 weeks post-conception significantly reduces the risk of severe language delay when tested in children at age 3. Did not find a relationship between maternal folic acid supplementation and delay in motor skills at age 3.	Self-reporting by mothers via questionnaires on their children.
Steenweg-DeGraaf [12]	Does maternal folate status alter child emotional and behavioral issues?	Mother-child pairs from a population-based cohort: ‘Generation R Study’	Participants from Generation R study. Mothers provided information on child emotional and behavioral problems at the age of 3.	Rotterdam, The Netherlands	Higher risk of emotional problems in offspring of mothers who were folate deficient but no effect on behavioral problems. The researchers report that homocysteine levels and MTHFR genotype were not associated with either behavioral or emotional problems.	Socioeconomic status and other factors of mothers enrolled varied and impact emotional and behavioral health of these children.
Chatzi [13]	Is there a relationship between high doses of folic acid supplementation in early pregnancy with offspring neurodevelopment at 18 months of age?	Mother-child cohort known as the ‘Rhea’ cohort	Women evaluated throughout the pregnancy with basic natal examinations and ultrasounds. Those who agreed to follow-up were recruited for the current study.	Heraklion, Crete, Greece	Researchers found that 5 mg supplementation of folic acid daily was associated with a 5-unit increase for receptive communication and a 3.5-unit increase for expressive communication when compared to no folic acid supplementation at all. Folic acid supplementation higher than 5 mg daily did not have a correlation with improved neurodevelopment.	The mothers self reported findings via telephone to interviewers who were assessing for offspring neurodevelopment.
Joubert [14]	Is there an association between maternal plasma folate during pregnancy and genome wide differential DNA methylation?	Newborns from 2 European Caucasian pregnancy cohorts: ‘Norwegian Mother and Child Cohort Study’ and the ‘Generation R study’	Children had their cord blood analyzed for DNA methylation measurements and maternal plasma folate was measured at 18 weeks gestation. The second group assessed for this study is the Generation R population, assessed maternal folate, Vitamin B12, homocysteine, Vitamin D, and SNP measurements. The children were assessed for DNA methylation via evaluation of cord blood.	Norway and Netherlands	There is an association between maternal plasma folate levels and DNA methylation in cord blood at 443 CpGs. The researchers also performed sensitivity tests and noted that Vitamin B12 does not “confound” the folate methylation association that they observed.	The researchers noted that the sex of the children in the study was not expected to be associated with maternal plasma folate and was not included in the analyses.
Henry [15]	Does folic acid supplementation in the second and third trimester in pregnancy affect child psychosocial health (emotional intelligence and resilience)?	Mother-child pairs from: ‘Folic Acid Supplementation in the Second and Third Trimester study’	Women that had taken folic acid supplementation throughout the first trimester of pregnancy and were recruited into the study at 14 weeks gestational age. Women were given either placebo or 400 μg/day of folic acid for 26 weeks.	Ireland	Mothers who received full-term supplementation of folic acid compared to mothers who only took supplementation through the end of the first trimester had children who scored significantly higher on the emotional intelligence and resilience tests. Maternal folate status at 36 weeks gestational age was a strong ‘predictor’ of emotional intelligence and resilience in offspring.	The sample size in this study was small, secondary to lack of funding as reported by the researchers.
Eryilmaz [16]	Does fetal folic acid exposure alter cortical maturation and psychiatric health in children?	Children from an observational clinical cohort study at Massachusetts General Hospital	Children recruited for this study were between 8 and 18 years of age at the time of the MRI scan, with birth dates between January 1993 and December 2001, as folic acid fortification was introduced to the US in 1996–1997.	USA	Folic acid exposure is associated with cortical thickness increase in the bilateral, frontal, and temporal regions, as well as delayed age-associated cortical thinning in the temporal and parietal regions.	MRI data collection was performed at different locations and with different MRI machines. This introduces a chance for error.
Huang [17]	Is there a relationship between maternal folate levels during pregnancy and children’s neuropsychological development at 2 years of age?	Children from ‘MKFOAD’ birth cohort in China	Subjects for this study were recruited from the MKFOAD cohort. The mothers had red blood cell and serum folate concentrations quantified at three different time periods while pregnant.	China	Maternal serum folate in late pregnancy was associated with children’s language development at age 2. The study also found that maternal serum folate in early pregnancy was inversely related to fine motor development in the children.	The researchers mentioned their own limitations in this study which includes testing a larger sample size.
Zou [18]	Is there a relationship between prenatal folate exposure and brain development in childhood?	Mother-child pairs from the ‘Generation R study’	The participants in this study were recruited were from the Generation R study. For this study specifically, inclusion requirements involved neuroimaging and exclusions were made based on MRI findings. Children aged 9–11 years old.	Rotterdam Netherlands	Maternal folate deficiency (accounted for as levels less than 7 nmol/L during pregnancy) resulted in lower offspring total brain volume and less cerebral white matter in children 9–11 years old. They did not note any differences in cortical thickness.	The participants for this study are derived from an already established cohort, which may introduce variables.

**Table 2:** Summary of main findings from pre-clinical studies.

Citation	Study Question(s) Being Investigated	Model System Used	Study Design	Main Findings	Limitations
Craciunescu [19]	Does maternal folate status during late gestation affect neurogenesis in the developing mouse brain?	Mouse	Pregnant mice at day 11 of gestation were assigned to either the folate-deficient group, control diet, or folate-supplemented group. The pregnant mice were euthanized on gestation day 17. Tissues were collected at end of the study.	Folate deficiency decreases the number of progenitor cells that divide in the developing mouse brain septum, caudate putamen, and the neocortex. In total, 106.2% more apoptotic cells were discovered with folate deficiency vs. in the control mouse brain. More calretinin-positive cells in the medial–septal diagonal band region of folate-deficient vs. control brains. Progenitor cells in the fetal brain are sensitive to folate status in late gestation.	Deficiency of other methyl donors was not excluded in this study, which may also contribute to the altered brain morphology.
Langie [20]	Does maternal folate depletion during pregnancy and lactation reduce DNA repair secondary to aberrant methylation and expression of base excision repair genes in the brain of adult mice offspring? Does postweaning exposure to high-fat diet enhance adverse effects of folate depletion?	Mouse	Adult female mice were assigned to a folate adequate group w/2 mg folic acid/kg diet or folate depleted group w/0.4 mg folic acid/kg diet. The diets were supplemented for 4 weeks before mating the male and female mice, and the diets continued through pregnancy and lactation. The pups were weaned at 22–25 days of age and assigned to either a control diet of 5% anhydrous milk or high-fat diet with 20% anhydrous milk fat (9 males and females in each group). At 5.5 months old, all animals were euthanized and tissue was analyzed.	Low-folate diet did not alter maternal body weight, litter size, or body weight of the offspring. High-fat diet postweaning resulted in increased body weight in the adult offspring but they did not attribute maternal folate supply to the adult offspring body mass.	The low sample size, which makes conclusions inconclusive and difficult to draw from.
Berrocal-Zarago [21]	What is the effect of a maternal folate-deficient diet on pup brain function?	Rats	Pregnant dams were fed a control diet. After birth, the dams and pups were separated into two groups, control and folic acid-deficient diets. The diets were started immediately after birth. At postnatal days 0 and 23, liver and brain tissue from dams and pups were collected for analysis. For the remaining pups, once they were separated they were continued on the control, low-folate, or high-folate diets. The pups were euthanized after behavioral testing, and the livers and brains were collected for analysis.	Developmental issues in the folate-deficient pups vs. the normal diet pups. There were also long-term memory and learning issues in the folate-deficient group. They found that folic acid supplementation in the post-weaning phase resulted in no change in the noted deficits. Maternal folate deficiency during weaning affects neurodevelopment of pups, even if folate status is sufficient in pregnancy.	The differences in myelination patterns in rats vs. humans in early life.
Jadavji [22]	Does maternal MTHFR deficiency alter offspring brain health? Does folate and choline dietary deficiency alter offspring brain function?	Mouse	Study 1: Maternal MTHFR deficiency. Female *Mthfr*^+/+^ and *Mthfr*^+/−^ mice were fed a control diet with 2 mg/kg folic acid, along with other nutrients. Study 2: Dietary deficiency study. Dams were fed either a control diet, folic acid-, or choline-deficient diet. Male offspring underwent behavioral testing and tissue collection for analysis.	Short-term memory issues in the offspring who had maternal MTHFR deficiency with elevated levels of homocysteine in the mothers, but no elevation in the offspring. Offspring from *Mthfr*^+/−^ mothers were noted to have increased cell death and growth in the hippocampus. For the maternal dietary deficiency study, there was also short-term memory issues and increased hippocampal cell death in the offspring of mothers who had folate and choline deficiency during pregnancy. There was noted to be increased neuron growth in the offspring of mothers who had choline deficiency during pregnancy.	A limitation of this study is the analysis of hippocampal and granular cell layers only.
Ly [23]	Which gestation period and organ is more sensitive to maternal folic acid on DNA methylation and gene expression in offspring?	Rats	Male and female rats were mated and maintained on a control diet. After confirmation of pregnancy, the dams were placed on 5 different folic acid diets: control, 2.5× the control diet for either 1st, 2nd, or 3rd week of gestation, or 2.5× the control throughout the entire pregnancy. Tissues were collected at the end of the study for analysis.	The brain is the most sensitive to maternal folic acid supplementation on global DNA methylation. The liver was sensitive to the effects of folic acid on gene expression in a gene-specific manner.	Other methyl donor deficiency is not considered in this study for altered DNA methylation status.
Barua [24]	Does excess maternal folic acid supplementation result in gene expression changes in the cerebral cortex of offspring?	Mouse	Female mice were divided into two groups prior to mating, and started on a control diet or excess folic acid. Tissues were collected at the end of study for analysis.	High maternal folic acid resulting in upregulation of the genes *Nfix*, *Runx1*, *and Vgll2* in female offspring but downregulation in male offspring. There was also increased expression of *Dnmt3b* but no altered expression of *Dnmt3a*. Imprinted gene *Dio3* was upregulated in male offspring dams with high maternal folic acid, but not female offspring. Imprinted genes *H19* and *Hist* were upregulated in female offspring from dams with high maternal folic acid. They also found decreased expression of *Auts2* (autism susceptible gene) in male offspring from dams with high maternal folic acid but increased expression in female offspring. *Park2* was upregulated in both male and female mice from dams with high maternal folic acid. DNA methylation levels were unaltered with high maternal folic acid.	Focus on histological analysis of the cerebral cortex only for altered gene expression, there were no behavioral tests conducted.
Bahous [25]	Does high maternal folic acid intake alter brain function and/or metabolism in offspring?	Mice	Female mice were placed on control or high-folic acid diets. These mice were sacrificed at day 17.5 of gestation while another group containing mice from both diet groups was maintained on the diets through both pregnancy and lactation. In brain tissue (hippocampus), folate and choline metabolites were measured.	Smaller hippocampal and dentate gyrus thickness size in pups of the mothers on the high-folate diet, as well as memory impairment. In pups of the mothers who had the high-folate diet, there were also decreased levels of MTHFR, as well as phosphocholine and glycerophosphocholine in the liver and hippocampus. There were also decreased acetylcholine and *Dnmt3a* levels in the hippocampus and cortex. Altered morphology of the embryo brain with altered cortical layer development.	One additional variable that could have been tested for this study is varying concentrations of folic acid instead of just 2× the amount of the control diet. This would have possibly shown what the cut off is for negative impacts of excessive folic acid intake.
Wang [26]	Is folic acid supplementation throughout pregnancy beneficial for offspring neurobehavioral development?	Mice	Four diet groups were included in this study: folate normal diet, folate-deficient diet, folate-supplemented diet short period, folate-supplemented diet long period. At days 4 to 8 postnatal neurobehavioral testing was conducted. Hippocampal tissue was collected from 4-month-old male offspring of the normal diet and folate-deficient long diet.	Increased plasma homocysteine levels in the folate-deficient group vs. the folate normal group, as well as impaired neurobehavioral development in offspring. This was represented by delayed sensory-motor reflex development in infancy, impaired spatial learning, and memory ability in adolescence and adulthood. There were also changes to the ultrastructure of the hippocampus in adulthood.	Focus on the length of time folic acid was supplemented, instead of also including varying levels of folic acid to determine the outcome on neurodevelopment.
Wu [27]	Does excessive folic acid supplementation affect β-catenin phosphorylation and *PP2Ac* methylation in an adult brain?	Mice	At 8 months of age, mice were divided into two groups: the control group was fed food and water without folic acid, and the experimental group was fed 2× the amount of folic acid. The mice were then mated, and folic acid supplementation continued in the water supply until postnatal day 21. Seven male offspring were killed at postnatal day 21, and 5 male offspring at 5 months for brain analysis.	Levels of non-phosphorylated β-catenin were increased in the brains of weaning and adult folic acid-exposed offspring; demethylation of *PP2Ac* was decreased, and *PI3K*, *Akt*, and *GSK3-β* were stimulated in the folic acid-exposed brains only at weaning. Excess folic acid supplementation may increase β-catenin via inhibition of *PP2Ac* demethylation, and indicate that this may be a mechanism for the role of folic acid on neurodevelopment.	Only male mice were used and there was small sample size.
De Crescenzo [28]	What are the effects of maternal folate deficiency or excess folate on neurodevelopment in offspring?	Mouse	Histological data for this study were obtained from embryos at day 14.5, and pups at postnatal day 0 or 6 from each diet group (control, folic acid-deficient, folic acid-supplemented). Behavioral testing was carried out in 4–10-week-old mice, and brain tissue was collected for analysis.	Maternal folic acid deficiency or an excess of it can result in an increase in late-born neurons compared to early-born neurons in the cerebral cortex. Increased apoptosis of deep layer neurons in both folic acid-deficient and folic acid-excess mice. Altered neuronal morphology when compared to control mice. Behavioral testing showed that pups of folic acid-deficient dams did not perform as well as the control in object recognition. Pups born to folic acid-excess dams revealed higher anxiety during the open maze test. Behavioral findings were correlated to changes in cortex morphology.	This study was not clear on the sample size used for the several different outcomes assessed, including histological analysis.
Liu [29]	Does maternal folic acid supplementation alter offspring adult metabolism?	Rats	Does maternal folic acid supplementation alter offspring adult metabolism?	Metabolic changes with maternal folic acid supplementation vs. no supplementation. The metabolic changes are reflected by the presence of phospholipids, fatty acids, and amino acids that are associated with linoleic acid, docosahexaenoic acid, glycerophosphocholine, lysophosphatidylcholine, tryptophan, glycine, arachidonic acid, and ƴ-aminobutyric acid. Fifty-one proteins were also identified in the brains of the pups from the dams who received folic acid supplementations vs. the control diet without supplementation. Offspring of the dams who received folic acid supplementation had a better level of memory compared to the pups of the dams who did not have supplementation.	One thing to note for this study is the number of metabolites and proteins produced in the results. There are a lot of data present for what was tested, but this may make conclusions a bit more difficult to draw from.
Garcez [30]	What is the effect of folic acid supplementation in adult offspring?	Rats	The dams were divided into 5 diet groups, including 4 divisions within the control diet and the folate-deficient diet. These diets continued throughout pregnancy and lactation. Female offspring were divided into 8 groups based on the dam’s diet. Behavioral testing was carried out, followed by sacrificing the mice for analysis of the prefrontal cortex and hippocampus.	Age-related memory impairment was decreased in the rats who had maternal folate supplementation. Folic acid supplementation was also able to decrease levels of TNF-α and IL-1β in the rats, which are cytokines associated with aging. Folic acid deficiency resulted in decreased levels of IL-4 in the hippocampus of 2-month-old rats. Aging and maternal folate deficiency also resulted in decreased levels of brain derived neurotrophic factor in the hippocampus and nerve growth factor from the prefrontal cortex. Folate supplementation also prevented this.	Unclear presentation of sample sizes.
Yang [31]	Does excessive folic acid preconception, during pregnancy, or during lactation affect the brain of female adult mice?	Mouse	At 8 weeks of age, mice were divided either into the control group with no folic acid supplementation, or the experimental group with folic acid supplementation in their water supply. After one week, the mice were mated and the supplementation continued throughout pregnancy and lactation. At postnatal day 21, brain tissue was collected for analysis. Behavioral testing was performed on 2-month-old mice.	In adult male mice there was increased anxiety, decreased exploratory activity, and decreased motor coordination and spatial memory. RNA sequencing and qRT-PCR revealed increased transcription of the following genes: *Tlr1*, *Sult1a1*, *Tph2, Acacb*, *Etnppl*, *Angplt4*, *and Apold1*; and decreased transcription of *Ppar-*α in the folic acid-exposed female brains. Excess folic acid supplementation during pregnancy and during lactation altered the brain transcriptome in weaning female mice, and alters adult female mice behavior. Behaviors in presented study were different from previous work.	The estrus cycle was not monitored in female mice during experiments.
